# Contrast-enhanced CT-based radiomics for predicting visceral pleural invasion in early-stage non-small cell lung cancer

**DOI:** 10.1186/s13244-025-02184-2

**Published:** 2026-01-26

**Authors:** Qinyue Luo, Hanting Li, Yuting Zheng, Yuting Lu, Lin Teng, Jun Fan, Xiaoyu Han, Heshui Shi

**Affiliations:** 1 Department of Radiology, Union Hospital, Tongji Medical College, Huazhong University of Science and Technology, Wuhan, China; 2 Hubei Provincial Clinical Research Center for Precision Radiology & Interventional Medicine, Wuhan, China; 3 Hubei Key Laboratory of Molecular Imaging, Wuhan, China; 4 Department of Medical Imaging, Henan Provincial People’s Hospital & People’s Hospital of Zhengzhou University, Zhengzhou, China; 5 Department of Pathology, Union Hospital, Tongji Medical College, Huazhong University of Science and Technology, Wuhan, China

**Keywords:** Non-small cell lung cancer, Visceral pleural invasion, Radiomics, Early-stage, Computed tomography

## Abstract

**Objectives:**

Waiting for postoperative pathologic confirmation of visceral pleural invasion (VPI) may delay treatment decisions. This study aimed to develop a contrast-enhanced CT-based radiomics model for preoperative prediction of VPI in early-stage non-small cell lung cancer (NSCLC).

**Materials and methods:**

We retrospectively enrolled 523 surgically resected NSCLC patients (195 with VPI, 328 without VPI) with clinically staged IA based on preoperative imaging between December 2019 and June 2022. Patients were randomly divided into training, validation, and testing sets at a ratio of 5:2:3. For each patient, 13 CT features were recorded, including the types I–V tumor relationships to the pleura. Regions of interest (ROIs) were segmented semi-automatically using deep learning. Least absolute shrinkage and selection operator (LASSO) regression was applied to select key radiomics features. Three models were developed: a CT-feature model, a radiomics model, and a combined model. The performance and clinical utility of these models were evaluated using the area under the curve (AUC) and decision curve analysis.

**Results:**

The tumor relationship to the pleura, density, maximum diameter, and spiculation were selected to construct the CT-feature model. A total of 10 optimal features formed the radiomics model. The radiomics model achieved an AUC of 0.812 in the testing set, outperforming the CT-feature model (0.714). Furthermore, the combined model showed a slightly higher AUC (0.825) compared to the radiomics model.

**Conclusions:**

The radiomics model demonstrated satisfactory performance for predicting VPI in early-stage NSCLC, outperforming the CT-feature model. The integration of radiomics and CT features may provide enhanced predictive value.

**Critical relevance statement:**

This study constructed a contrast-enhanced CT-based radiomics model with promising performance for the preoperative prediction of VPI, which aims to guide treatment planning for early-stage NSCLC.

**Key Points:**

- VPI affects the tumor-node-metastasis (TNM) staging of tumors and subsequent treatment strategies.
- The radiomics model outperformed the CT-feature model in predicting VPI.
- The contrast-enhanced CT-based radiomics model may be valuable for optimizing clinical decision-making.

**Graphical Abstract:**

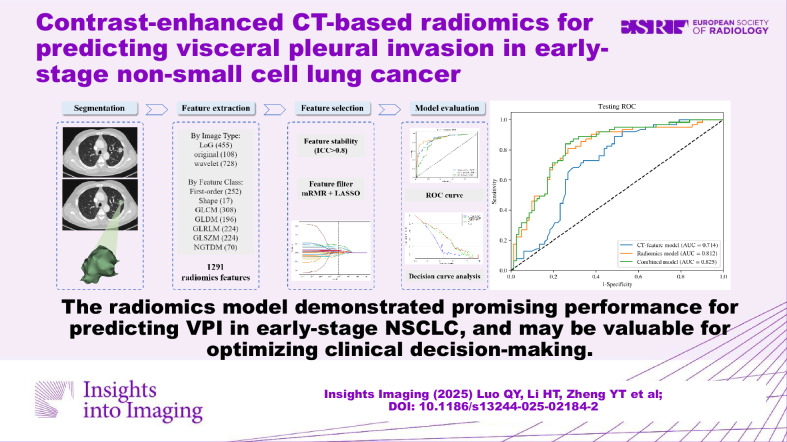

## Introduction

Lung cancer is the leading cause of cancer-related death [1]. It is important to know if there is visceral pleural invasion (VPI) because it has a direct effect on how to stage tumors and how to treat them [2]. Early-stage non-small cell lung cancer (NSCLC) without VPI usually requires segmental surgical resection, whereas lobectomy is typically performed if VPI is present [3, 4]. VPI is a high-risk pathological factor for recurrence, and patients with VPI may benefit from postoperative adjuvant chemotherapy [5]. However, postoperative pathological confirmation of VPI may delay decisions on the surgical approach and adjuvant therapy.

VPI is associated with nodule density, maximum tumor diameter, and the bridge tag sign [6–8]. Several studies have found a closer association between VPI and the tumor-pleura relationship on CT [9–11]. Hsu et al suggested that type 2 pleural tags could achieve an accuracy of 71% in the early diagnosis of VPI [9]. However, Kim et al reported that the positive predictive value of CT features for diagnosing VPI ranged from 44.1% to 56.4%, with nearly half of the CT-based VPI predictions being false positives [11]. Accurately diagnosing VPI in NSCLC based solely on CT features remains challenging.

Radiomics analyzes quantitative imaging features, including texture patterns and spatial relationships, to support lung cancer management through feature selection and model establishment, aiding in diagnosis, treatment evaluation, and outcome prediction [12–15]. Several studies [16, 17] have developed models to predict VPI, and the areas under the receiver operating characteristic curves (AUCs) for their radiomics models were 0.824 and 0.771, respectively. However, Wei et al used 2D images for feature extraction, and Cai et al included only solid nodules in their study, both with small sample sizes. Considering these limitations, this study aimed to evaluate the value of a CT-based radiomics model for preoperative VPI prediction in early-stage NSCLC, while also developing a CT-feature model that incorporates tumor-pleura relationships and constructing a combined model to compare their predictive performances.

## Materials and methods

The study was conducted in accordance with the 2013 revision of the Declaration of Helsinki. The study was approved by the Ethics Committee of Wuhan Union Hospital (No. S254), and informed consent for this retrospective analysis was waived.

### Patient enrollment

**Fig. 1:**
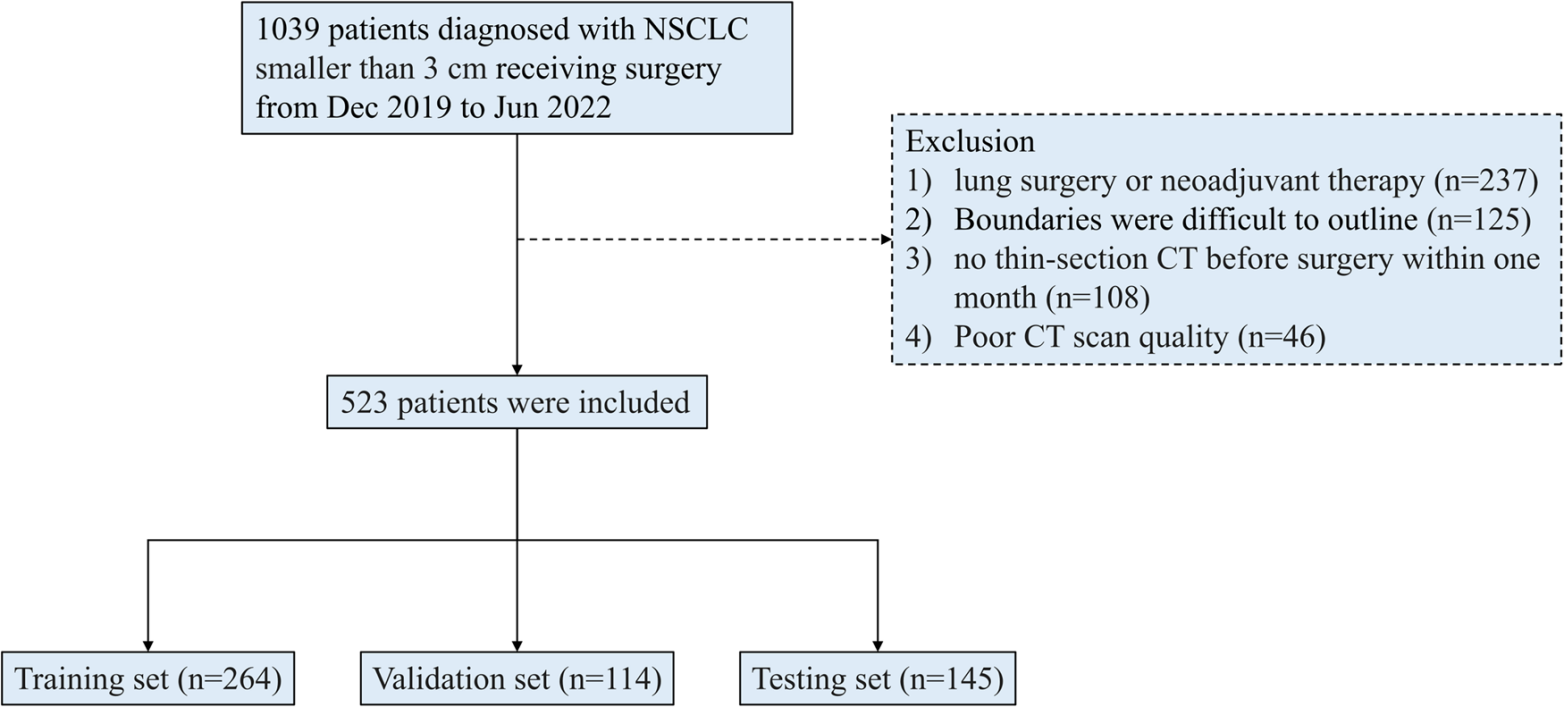
The flow diagram shows patient selection and exclusion criteria. NSCLC, non-small cell lung cancer; CT, computed tomography

A total of 1039 patients with NSCLC smaller than 3 cm, diagnosed by surgery between December 2019 and June 2022 at Wuhan Union Hospital, were retrospectively collected. All patients were clinically staged as IA based on preoperative imaging according to the International Association for the Study of Lung Cancer (IASLC) 8th edition TNM classification. VPI status was confirmed by pathological examination after surgery, and some tumors may have been upstaged to pathological stage IB. The inclusion criteria were as follows: [1] surgically resected pathologically confirmed NSCLC; [2] tumor less than 3 cm; [3] without lymph node metastasis and distant metastasis; [4] less than one month between surgery and last preoperative CT examination; [5] specific elastic staining to assess the presence of VPI; [6] all tumors were subpleural and exhibited at least one type of tumor relationship to the pleura. Of these, 516 patients were excluded based on the following exclusion criteria: [1] patients with previous oncologic lung surgery or neoadjuvant therapy (*n* = 237); [2] tumors with unclear boundaries that were difficult to outline (*n* = 125); [3] no thin-section CT before surgery within one month (*n* = 108); [4] the quality of CT imaging was poor, defined as the presence of motion artifacts that obscured lesion borders and interfered with accurate tumor delineation (*n* = 46). The flowchart of patient selection was shown in Fig. 1. Finally, 523 patients (195 with VPI and 328 without VPI) were included and randomly divided into a training set (264 cases), a validation set (114 cases), and a testing set (145 cases) in the ratio of 5:2:3. The validation set was used to avoid model overfitting and tune hyperparameters, while the testing set was used to assess model performance and generalizability. Clinical characteristics, including age, gender, and smoking history, were retrieved from the patients’ medical records.

### Histopathological analysis

An experienced pathologist (J.F., with 12 years of experience in pathology), blinded to the clinical findings, re-evaluated the specific elastic staining specimens of all included patients. VPI was defined as cancer tissue penetrating the elastic layer, including invasion to the visceral pleural surface [18].

### CT acquisition

All patients underwent contrast-enhanced chest CT scans before surgery, and the CT was performed on two multislice spiral CT scanners (SOMATOM Definition AS+, Siemens Healthineers, and Toshiba Aquilion One). Detailed information of the CT parameters was as follows: detector collimation widths, 64 × 0.6 mm and 128 × 0.6 mm; tube voltages, 120 kV. The tube current was regulated by an automatic exposure control system (CARE Dose 4D). Nonionic iodine contrast medium (iohexol 350 mg/mL, Beilu Pharmaceutical Co., Ltd.) was injected intravenously. The rate of injection was 2–3 mL/s. A slice thickness of 1.5 mm or 1 mm and an interval of 1.5 mm or 1 mm were used to reconstruct the images. Digital imaging and communication system in medicine (DICOM) images were retrieved from the picture archiving and communication system (PACS) and subsequently imported into the open-source 3D Slicer software (version 5.2.2; https://www.slicer.org) for further analysis.

### Image evaluation

**Fig. 2:**
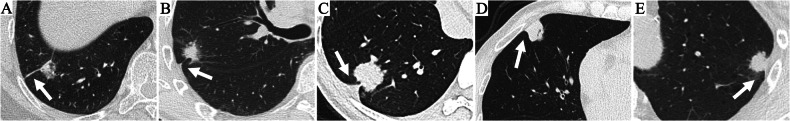
Five types of tumor relationships to the pleura. **A** Type I, one or more linear pleural tags (white arrow). **B** Type II, one or more linear pleural tags with soft tissue components at the pleural end (white arrow). **C** Type III, one or more bold-wire pleural tags with soft tissue components at the pleural end (white arrow). **D** Type IV, tumor attached to the pleura without obvious distortion (white arrow). **E** Type V, tumor in direct contact with the pleura, causing pleura distortion (white arrow)

Two professional radiologists (H.S. and X.H., with 35 and 8 years of experience in chest radiology, respectively) evaluated all the CT images in consensus on the PACS. The CT morphological characteristics assessed included: tumor maximum diameter (in lung window), tumor density type [solid, mixed ground-glass opacity (mGGO), and pure ground-glass opacity (pGGO)], five types of tumor relationships to the pleura (type I: one or more linear pleural tags; type II: one or more linear pleural tags with soft tissue components at the pleural end; type III: one or more bold-wire pleural tags with soft tissue components at the pleural end; type IV: tumor attached to the pleura without obvious distortion; type V: tumor in direct contact with pleura, causing pleura distortion; Fig. 2), involved pleura (non-interlobar fissure pleura, interlobar fissure pleura, and both), the presence of a solid portion in contact with the pleura, tumor location, spiculation, lobulation, lymphadenopathy, cavity, pleural thickening, pleural effusion, and calcification. The two radiologists evaluated CT features on axial and multiplanar reconstruction images and were blinded to clinical and pathologic findings.

### Tumor segmentation and radiomics feature extraction

Two junior radiologists (H.L. and Q.L.) performed semi-automatic segmentation of the regions of interest (ROIs) on CT images, tracing lesion boundaries while excluding vascular structures and calcification. We randomly selected 100 CT images from our dataset and outlined them using 3D Slicer software. The two radiologists, while informed about the tumor’s location, had no access to any clinical or pathological diagnostic information. A Residual U-Net with MONAI was then trained using these images, which were randomly split into training and validation sets in an 8:2 ratio [19, 20]. After applying the trained Residual U-NET model, the remaining images were segmented automatically. To guarantee accuracy, two senior radiologists checked and adjusted the automatically segmented ROIs. Moreover, intraclass correlation coefficients (ICCs) were used to assess segmentation accuracy. One radiologist (Q.L.) manually segmented 50 randomly selected CT images using 3D Slicer. An ICC cutoff value of > 0.8 was utilized to identify stable and reproducible features.

**Fig. 3:**
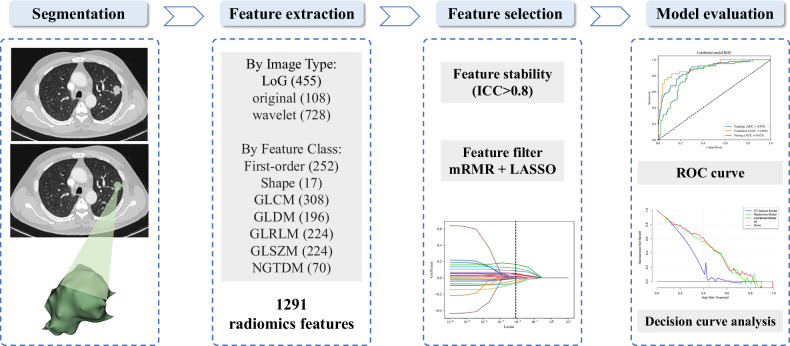
Radiomics workflow

Radiomics features were automatically extracted from ROIs using the PyRadiomics package (version 3.0.1; https://pyradiomic.readthedocs.io/en/v3.0.1/). Extraction was performed on the arterial phase of contrast-enhanced CT images, which provides clear tumor-pleura contrast and consistent image quality. The following parameters were applied: the imaging data underwent normalization using mean standard deviation centering, followed by B-spline interpolation resampling to achieve 1 × 1 × 1 mm^3^ voxel dimensions, with gray-level discretization standardized at 25 HU bin width [21]. A total of 1291 radiomics features were retrieved from each ROI (Fig. 3), including 455 loG features, 108 original features, and 728 wavelet features. These features covered various categories: 252 first-order features, 17 shape features, 308 gray-level co-occurrence matrix (GLCM) features, 196 gray-level dependency matrix (GLDM) features, 224 gray-level run length matrix (GLRLM) features, 224 gray-level size zone matrix (GLSZM) features, and 70 neighborhood gray-tone difference matrix (NGTDM) features.

### Feature selection and model building

We followed a three-step procedure to reduce dimensionality and select robust radiomics features within the training set. First, the extracted radiomics feature values were normalized using the *Z*-score. Second, the maximum relevance minimum redundancy (mRMR) and Spearman’s rank correlation coefficient method were employed to eliminate irrelevant and redundant variables. Finally, a 10-fold cross-validation least absolute shrinkage and selection operator (LASSO) analysis was performed to identify the best radiomics features [21].

A multivariate logistic regression (LR) algorithm was used to develop the classification model based on the selected features. The CT-feature model was constructed using significant CT characteristics that differentiated the two groups, while the radiomics model was built from the most influential radiomics features. Finally, a combined model was developed by integrating the CT features with the RadScore. Additionally, we used support vector machine (SVM), K-nearest neighbors (KNN), decision tree (DT), random forest (RF), and XGBoost machine learning algorithms to construct models. The optimal machine learning algorithm was selected based on the evaluation of both fitting performance and generalization performance.

### Statistical analysis

Statistical analyses were performed using SPSS software (SPSS, version 26, IBM, Chicago, IL, USA) and R software (version 4.3.3, available at http://www.rproject.org/). Continuous variables were presented as medians with interquartile ranges (IQR), and categorical variables as frequencies with percentages. The Mann–Whitney *U*-test was used for continuous variables, while Chi-square or Fisher’s exact tests were applied for categorical variables. We calculated the Kappa value and ICC to assess the consistency between the two readers (H.S. and X.H.). The performance of the proposed models was evaluated by plotting receiver operating characteristic (ROC) curves and calculating the corresponding AUCs. The Delong test was applied to compare diagnostic performance between models. Additionally, decision curve analysis (DCA) was used to assess the clinical utility of each model. LASSO regression was performed with the ‘glmnet’ package, ROC analysis with ‘pROC’, multivariate LR and calibration with ‘rms’, and DCA with ‘rmda’. A two-tailed *p* value of <0.05 was considered statistically significant.

## Results

### Clinical characteristics and CT features

A total of 523 eligible patients (195 with VPI and 328 without VPI) were included in this study. With the exception of density (*p* = 0.013), there were no statistically significant differences in any other baseline characteristics of the training, validation, and testing sets (Table S1). As shown in Table 1, VPI was less prevalent in males (50/92 vs 64/172, *p* = 0.007) in the training set. In the validation set, patients with VPI were older (63 years vs 57 years, *p* = 0.001). No statistically significant differences in age, gender, or smoking history were found between the VPI and non-VPI groups in the testing set (all *p* > 0.05).

**Table 1:** Clinical characteristics and CT features in the training, validation, and testing sets

	Training set		*p* value	Validation set		*p* value	Testing set		*p* value
	VPI (+)	VPI (−)		VPI (+)	VPI (−)		VPI (+)	VPI (−)	
	(*n* = 92)	(*n* = 172)		(*n* = 40)	(*n* = 74)		(*n* = 63)	(*n* = 82)	
Gender (male)	50 (54.3%)	64 (37.2%)	0.007^*^	21 (52.5%)	29 (39.2%)	0.172	32 (50.8%)	35 (42.7%)	0.332
Age (years), median (IQR)	60 [54, 65]	58 [51, 65]	0.336	63 [58, 70]	57 [51, 65]	0.001^*^	63 [55, 67]	60 [51, 66]	0.147
Smoking	23 (25.0%)	39 (22.7%)	0.671	9 (22.5%)	11 (14.9%)	0.306	16 (25.4%)	16 (19.5%)	0.397
Relationship to the pleura			0.004^*^			0.136			0.043^*^
Type I	7 (7.6%)	34 (19.8%)		1 (2.5%)	11 (14.9%)		3 (4.8%)	14 (17.1%)	
Type II	14 (15.2%)	24 (14.0%)		8 (20.0%)	11 (14.9%)		8 (12.7%)	11 (13.4%)	
Type III	17 (18.5%)	10 (5.8%)		4 (10.0%)	7 (9.5%)		9 (14.3%)	6 (7.3%)	
Type IV	8 (8.7%)	14 (8.1%)		4 (10.0%)	14 (18.9%)		4 (6.3%)	12 (14.6%)	
Type V	46 (50.0%)	90 (52.3%)		23 (57.5%)	31 (41.9%)		39 (61.9%)	39 (47.6%)	
Density			< 0.001^*^			< 0.001^*^			< 0.001^*^
pGGO	2 (2.2%)	27 (15.7%)		0 (0.0%)	15 (20.3%)		2 (3.2%)	16 (19.5%)	
mGGO	34 (37.0%)	117 (68.0%)		11 (27.5%)	50 (67.6%)		14 (22.2%)	44 (53.7%)	
solid	56 (60.9%)	28 (16.3%)		29 (72.5%)	9 (12.2%)		47 (74.6%)	22 (26.8%)	
Maximum diameter(mm)	21 [18, 24]	17 [13, 21]	< 0.001^*^	22 [19, 26]	18 [15, 21]	< 0.001^*^	23 [18, 25]	17 [12, 22]	< 0.001^*^
Spiculation	64 (69.6%)	66 (38.4%)	< 0.001^*^	27 (67.5%)	32 (43.2%)	0.013^*^	40 (63.5%)	37 (45.1%)	0.028^*^
Lobulation	45 (48.9%)	28 (16.3%)	< 0.001^*^	21 (52.5%)	12 (16.2%)	< 0.001^*^	33 (52.4%)	15 (18.3%)	< 0.001^*^
Lymphadenopathy	16 (17.4%)	12 (7.0%)	0.009^*^	5 (12.5%)	3 (4.1%)	0.126	10 (15.9%)	11 (13.4%)	0.677
Presence of a solid portion in contact with the pleura	55 (59.8%)	78 (45.3%)	0.025^*^	26 (65.0%)	28 (37.8%)	0.006^*^	44 (69.8%)	33 (40.2%)	< 0.001^*^
Tumor location			0.386			0.831			0.543
Left upper lobe	18 (19.6%)	45 (26.2%)		10 (25.0%)	15 (20.3%)		12 (19.0%)	13 (15.9%)	
Left lower lobe	10 (10.9%)	22 (12.8%)		4 (10.0%)	7 (9.5%)		11 (17.5%)	14 (17.1%)	
Right upper lobe	37 (40.2%)	50 (29.1%)		13 (32.5%)	32 (43.2%)		18 (28.6%)	25 (30.5%)	
Right middle lobe	12 (13.0%)	20 (11.6%)		2 (5.0%)	3 (4.1%)		10 (15.9%)	7 (8.5%)	
Right lower lobe	15 (16.3%)	35 (20.3%)		11 (27.5%)	17 (23.0%)		12 (19.0%)	23 (28.0%)	
Involved pleura			0.106			0.026^*^			0.037^*^
Non-interlobar fissure pleura	60 (65.2%)	102 (59.3%)		24 (60.0%)	47 (63.5%)		36 (57.1%)	49 (59.8%)	
Interlobar fissure pleura	14 (15.2%)	45 (26.2%)		4 (10.0%)	18 (24.3%)		6 (9.5%)	18 (22.0%)	
both	18 (19.6%)	25 (14.5%)		12 (30.0%)	9 (12.2%)		21 (33.3%)	15 (18.3%)	
Cavity	9 (9.8%)	15 (8.7%)	0.775	4 (10.0%)	3 (4.1%)	0.238	4 (6.3%)	11 (13.4%)	0.166
Pleural thickening	47 (51.1%)	71 (41.3%)	0.127	25 (62.5%)	26 (35.1%)	0.005^*^	43 (68.3%)	30 (36.6%)	< 0.001^*^
Pleural effusion	0 (0.0%)	0 (0.0%)	NA	0 (0.0%)	0 (0.0%)	NA	1 (1.6%)	0 (0.0%)	0.434
Calcification	1 (1.1%)	5 (2.9%)	0.668	1 (2.5%)	0 (0.0%)	0.351	5 (7.9%)	1 (1.2%)	0.086

^*^ *p* value < 0.05

*VPI* visceral pleural invasion, *IQR* interquartile range, *pGGO* pure ground-glass opacity, *mGGO* mixed ground-glass opacity

Relationship to the pleura was significantly associated with VPI status in both the training (*p* = 0.004) and testing sets (*p* = 0.043). In the training set, type I was present in 7.6% and type V in 50.0% of the VPI group, with a similar trend observed in the testing set. However, no statistical difference was found for the relationship to the pleura in the validation set (*p* = 0.136). Univariate analysis showed significant differences (all *p* < 0.05) between the two groups in CT morphological features, including tumor density, maximum diameter, spiculation, lobulation, and the presence of a solid portion in contact with the pleura. Notably, the VPI group exhibited a higher frequency of solid nodules, larger sizes, and more lobulation. Other CT signs, such as tumor location, cavity, pleural effusion, and calcification, did not show any significant differences between the two groups (all *p* > 0.05). Inter-reader agreement was good for CT features, with kappa values ranging from 0.775 to 0.886 (Table S2). The ICC for maximum diameter was 0.940 [95% confidence interval (CI): 0.891–0.967].

**Fig. 4:**
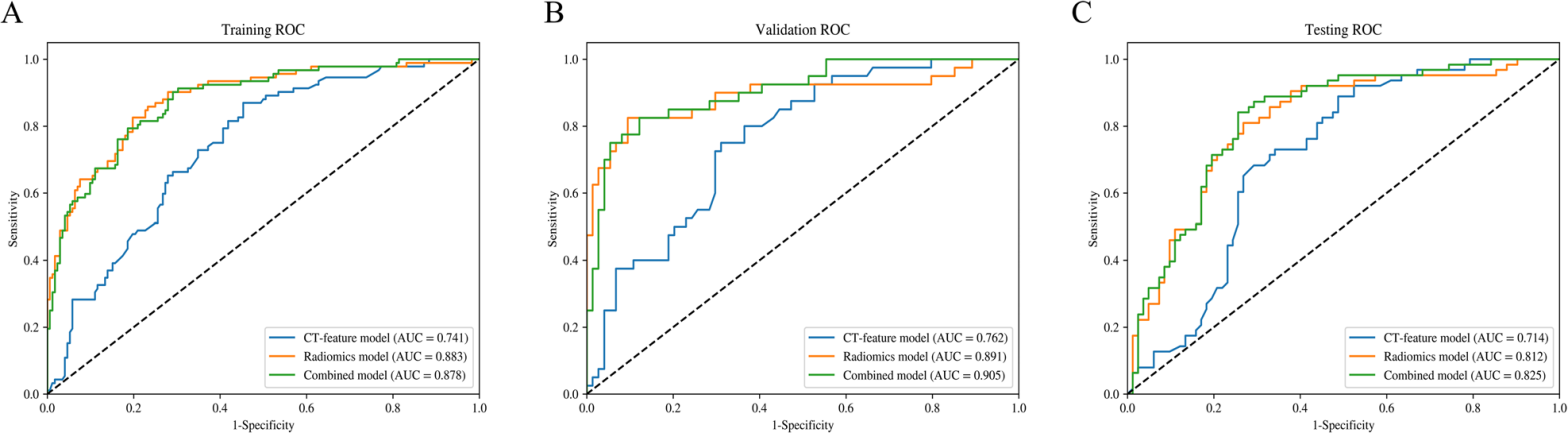
ROC curves for the prediction of VPI of the CT-feature model, radiomics model, and combined model in the training (**A**), validation (**B**), and testing (**C**) sets. ROC, receiver operating characteristic; VPI, visceral pleural invasion

A multivariate LR analysis was performed to incorporate tumor relationship to the pleura, along with density, maximum diameter, and spiculation, into the CT-feature model for predicting VPI in early-stage NSCLC. The model achieved AUCs of 0.741, 0.762, and 0.714 in the training, validation, and testing sets, respectively (Fig. 4 and Table S3).

### Radiomics model building and validation

**Fig. 5:**
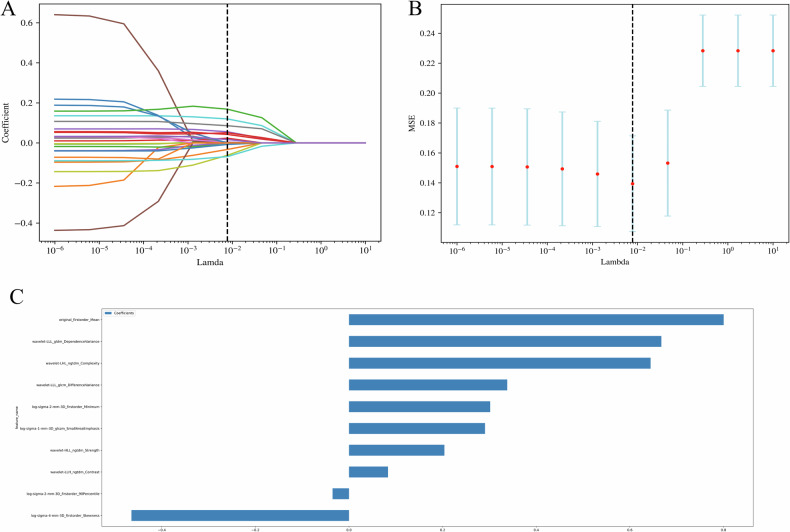
LASSO analysis of radiomics features (**A**) and the regularization parameter λ (**B**). **C** The feature weights of selected radiomics features. LASSO, least absolute shrinkage and selection operator

The best 14 radiomics features for VPI prediction were selected using the mRMR and Spearman’s rank correlation coefficient method. Next, 10 features (four first-order and six second-order, including GLCM, GLDM, NGTDM, and GLSZM) with non-zero coefficients in the training set were identified through LASSO analysis and used to build the radiomics model (Fig. 5). The model achieved AUCs of 0.883 in the training set, 0.891 in the validation set, and 0.812 in the testing set, demonstrating strong performance (Fig. 4).

The following formula was used to calculate the RadScore of each lesion:

RadScore = −0.86309 + 0.18 × wavelet-HLL_ngtdm_Strength + 0.19 × log-sigma-2-mm-3D_firstorder_Minimum + 0.57 × wavelet-LLL_gldm_DependenceVariance + 0.27 × log-sigma-1-mm-3D_glszm_SmallAreaEmphasis + 0.51 × wavelet-LHL_ngtdm_Complexity-0.35 × log-sigma-4-mm-3D_firstorder_Skewness-0.08 × log-sigma-2-mm-3D_firstorder_90Percentile + 0.66 × original_firstorder_Mean + 0.31 × wavelet-LLL_glcm_DifferenceVariance + 0.02 × wavelet-LLH_ngtdm_Contrast

### Performance comparison

The optimal CT features and radiomics features were integrated to develop a combined model. With AUCs of 0.878 (95% CI: 0.835–0.921) in the training set, 0.905 (95% CI: 0.847–0.963) in the validation set, and 0.825 (95% CI: 0.757–0.893) in the testing set, the combined model outperformed the CT-feature model in diagnostic efficiency (Fig. 4). The performance metrics for the three models in predicting VPI in early-stage NSCLC are shown in Table 2. The Delong test revealed significant performance differences in the training set, with the radiomics model (*p* < 0.001) and combined model (*p* < 0.001) both outperforming the CT-feature model. However, no significant differences were observed between the radiomics and combined models across all sets (*p* > 0.05). The predictive performance by different machine learning algorithms of three models on the testing set is presented in Table 3. The SVM, DT, and XGBoost algorithms showed a trend of overfitting across all three datasets. While the RF algorithm outperformed LR on the CT-feature model (AUC: 0.807 vs 0.714), the AUCs on the radiomics and combined models were lower (0.802 and 0.799, respectively). These results demonstrated that the LR model was the optimal choice for predicting VPI preoperatively.

**Table 2:** Confounder matrix for the performance of the three models in predicting VPI

Models	AUC	Accuracy (%)	Sensitivity (%)	Specificity (%)
CT-feature model				
Training set	0.741	65.9	87.0	54.7
Validation set	0.762	71.1	75.0	68.9
Testing set	0.714	67.6	88.9	51.2
Radiomics model				
Training set	0.883	81.1	82.6	80.2
Validation set	0.891	87.7	82.5	90.5
Testing set	0.812	76.6	81.0	73.2
Combined model				
Training set	0.878	77.7	90.2	70.9
Validation set	0.905	86.0	82.5	87.8
Testing set	0.825	78.6	84.1	74.4

*VPI* visceral pleural invasion, *AUC* area under the curve

**Table 3:** Prediction performance of CT-feature, radiomics, and combined models by different machine learning methods

Models	Algorithms	AUC (95% CI)	Accuracy	Sensitivity	Specificity
CT-feature model	LR	0.714 [0.629, 0.798]	0.676	0.889	0.512
	SVM	0.674 [0.583, 0.765]	0.690	0.698	0.683
	KNN	0.760 [0.682, 0.838]	0.703	0.921	0.537
	DT	0.682 [0.594, 0.770]	0.655	0.698	0.622
	RF	0.807 [0.734, 0.880]	0.766	0.857	0.695
	XGBoost	0.751 [0.672, 0.831]	0.710	0.873	0.585
Rdiomics model	LR	0.812 [0.741, 0.884]	0.766	0.810	0.732
	SVM	0.773 [0.696, 0.850]	0.738	0.667	0.793
	KNN	0.786 [0.710, 0.863]	0.779	0.794	0.768
	DT	0.691 [0.603, 0.778]	0.690	0.810	0.598
	RF	0.802 [0.728, 0.875]	0.772	0.810	0.744
	XGBoost	0.791 [0.719, 0.864]	0.745	0.825	0.683
Combined model	LR	0.825 [0.757, 0.893]	0.786	0.841	0.744
	SVM	0.765 [0.685, 0.845]	0.752	0.714	0.780
	KNN	0.805 [0.734, 0.877]	0.779	0.810	0.756
	DT	0.620 [0.522, 0.718]	0.669	0.651	0.683
	RF	0.799 [0.726, 0.872]	0.759	0.825	0.707
	XGBoost	0.816 [0.748, 0.884]	0.766	0.841	0.707

*LR* logistic regression, *SVM* support vector machine, *KNN* K-nearest neighbors, *DT* decision tree, *RF* random forest, *AUC* area under the curve

### Calibration analysis and clinical use

**Fig. 6:**
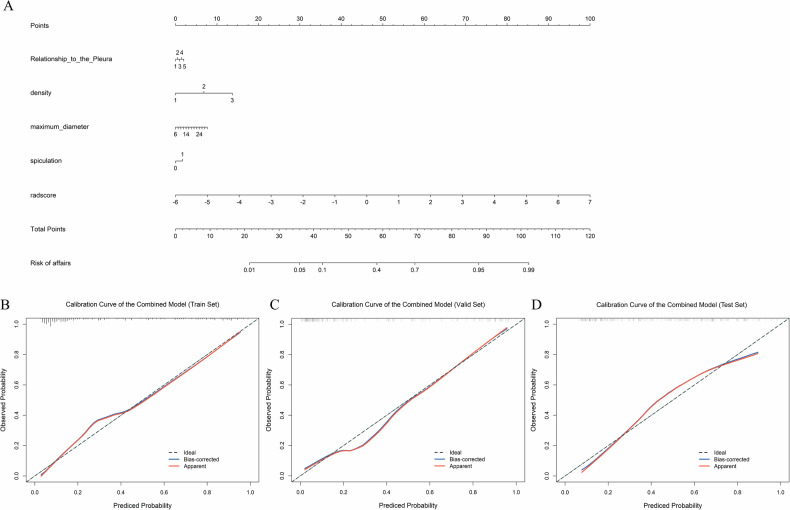
**A** Nomogram of the combined model for predicting VPI (relationship to the pleura: 1–5 = Types I–V; Density: 1 = pGGO, 2 = mGGO, 3 = solid; Spiculation: 0 = no, 1 = yes). **B**–**D** Calibration curves for the combined model in the training, validation, and testing sets. VPI, visceral pleural invasion

For better clinical application and visualization, we developed a nomogram based on the output of the combined model. This nomogram incorporated the four CT features and the RadScore, offering a more intuitive tool for VPI prediction (Fig. 6). The calibration curves of the combined model showed good accordance between the predictions and actual observed values (Fig. 6). We evaluated the clinical utility of the three models using DCA (Fig. S1). Both the radiomics and combined models provided greater net benefit than the CT-feature model.

## Discussion

In this study, we developed a contrast-enhanced CT-based radiomics model that demonstrated strong performance in predicting VPI in early-stage NSCLC. This model notably outperformed the CT-feature model, which relied on CT morphological features. Additionally, the combined model showed a marginally higher AUC than the radiomics model. This suggested that radiomics features may have already captured most of the discriminatory information provided by traditional CT features. Many traditional CT descriptors, such as margin, and lobulation, may be reflected in the high-dimensional radiomic texture and shape features. Therefore, the incremental contribution of adding CT features was limited. The radiomics model provides a reliable predictive method for early-stage NSCLC patients when making preoperative decisions.

In the present study, tumor relationship to the pleura was a risk factor for VPI, similar to the previous studies [9, 17]. Types III (one or more bold-wire pleural tags with soft tissue components at the pleural end) and IV (tumor attached to the pleura without obvious distortion) showed higher ORs, suggesting that pleural tags [9] and the length of tumor-pleura contact [22] are predictive factors for VPI. Type V (tumor in direct contact with the pleura and causing the pleura distortion) was frequently accompanied by marked pleural indentation. In line with the findings of Huang et al [23], pleural indentation reflected a higher degree of malignancy and an increased likelihood of VPI. Localized edema in the interlobular septum, tumor spreading along lymphatic vessels, inflammatory response, or fibrosis could be the pathologic basis of pleural tags [8, 9]. There is an increased risk of invasion of the visceral pleura as a result of the development of tumor-reactive fibroplasia and infiltrative spread of the tumor, which causes depressions and the extension of areas of depressions in the adjacent pleura. Clinically, these classifications may assist in preoperative risk stratification and surgical planning, particularly when VPI influences staging or resection margins. Lobectomy is generally recommended for improved prognosis when VPI is predicted [24].

Our results demonstrated that density was significantly associated with VPI status, with VPI occurring more frequently in solid nodules. Compared with pGGO, solid nodules showed an 8.8-fold higher risk of VPI, while mGGO also exhibited a trend toward increased risk. This finding aligns with the biological behavior that tumor aggressiveness increases progressively with the proportion of solid components [6, 23, 25]. Additionally, we found a strong correlation between large tumor size, spiculation, and VPI. These characteristics, as described in earlier studies [6, 7], suggested that the tumors were more aggressive. The patients in this study did not have lymph node metastases, and lymph node enlargement was less commonly observed, so lymphadenopathy was not included as a predictive feature of VPI in the model.

In our study, the radiomics approach showed promising results in predicting the presence of VPI in early-stage NSCLC. First-order features described the gray-scale distribution of the tumor, such as original_firstorder_Mean and log-sigma-4-mm-3D_firstorder_Skewness, which reflect variations in the tumor’s density, brightness, and heterogeneity [26]. Higher-order features, such as wavelet-HLL_ngtdm_Strength and wavelet-LLL_gldm_DependenceVariance, captured more complex texture patterns, effectively describing the tumor’s structural complexity and gray-level variations [27]. Together, these features reflected the heterogeneity and aggressiveness of the tumor, suggesting an increased risk of visceral pleura invasion.

ROC curve analysis in this study showed that the radiomics model was superior to the CT-feature model. This highlights the added value of radiomics features in predicting VPI. Wei et al developed a radiomics model based on texture features from 2D data, achieving an AUC of 0.824 [16]. Unlike the study by Wei et al, our 3D ROI analysis captured the spatial heterogeneity of tumors. Moreover, whereas Wei et al included NSCLC patients with lymph node metastases and used elastic fiber staining only when HE staining was inconclusive for pleural invasion, our study excluded patients with lymph node metastases and applied specific elastic staining in all cases. Cai et al employed Variance Thresholding and SelectKBest for feature selection and developed a radiomics model with an AUC of 0.771, whereas we applied mRMR combined with Spearman analysis and achieved an AUC of 0.812 [17]. Beyond that, the number of patients in this study was double the size of the two studies mentioned above. Radiomics may provide more insights into VPI.

The combined model achieved accurate VPI prediction by integrating CT features and radiomics features. The robustness of the findings was strengthened by the balanced distribution of baseline characteristics across datasets. Although nodule density differed significantly between sets, its limited absolute variation and minimal impact on model performance (e.g., consistent AUC > 0.80 in validation and testing sets) suggested that this imbalance was unlikely to compromise our conclusions. The comparability of all other key variables (e.g., tumor size) supports fair cross-dataset evaluation. Among the machine learning algorithms tested, the LR model demonstrated superior performance, likely reflecting the linear separability of the main predictive features in this cohort, its resistance to overfitting, and its interpretability in clinical practice.

The study had several limitations. Initially, due to the lack of an external validation cohort, our study was unable to fully assess the stability and generalizability of the radiomics model’s features. Secondly, long-term follow-up assessing survival and postoperative prognosis between groups was not conducted. Thirdly, the extent of pleural invasion was not evaluated. The extent of pleural invasion has been shown to result in different survival outcomes [18], and future research with standardized PL1-3 annotations may facilitate the development of tiered radiomics models capable of predicting both the presence and extent of pleural invasion, thereby refining adjuvant therapy decisions. Finally, the retrospective design introduced an inherent selection bias, and allowing up to one month between CT and surgery may have led to tumor progression or feature instability.

In summary, the contrast-enhanced CT-based radiomics model demonstrated promising diagnostic performance for preoperative VPI prediction, potentially serving as a valuable tool for treatment planning in early-stage NSCLC. Furthermore, the radiomics model outperformed the CT-feature model, demonstrating the added value of radiomics features in predicting VPI. However, further prospective multicenter studies are needed to validate these findings.

## Supplementary information

ELECTRONIC SUPPLEMENTARY MATERIAL

## Data Availability

The data is available from the corresponding author on reasonable request.

## Abbreviations

AUC: Area under the curve
CI: Confidence interval
DCA: Decision curve analysis
DT: Decision tree
GLCM: Gray-level co-occurrence matrix
GLDM: Gray-level dependency matrix
GLSZM: Gray-level size zone matrix
ICC: Intraclass correlation coefficient
KNN: K-nearest neighbors
LASSO: Least absolute shrinkage and selection operator
LR: Logistic regression
mGGO: Mixed ground-glass opacity
mRMR: Maximum relevance minimum redundancy
NGTDM: Neighborhood gray-tone difference matrix
NSCLC: Non-small cell lung cancer
PACS: Picture archiving and communication system
pGGO: Pure ground-glass opacity
RF: Random forest
ROC: Receiver operating characteristic
ROI: Region of interest
SVM: Support vector machine
VPI: Visceral pleural invasion
