# Supplementary material for: Contrast-enhanced CT-based radiomics for predicting visceral pleural invasion in early-stage non-small cell lung cancer
Source: Insights Imaging. 2026 Jan 26;17:17. doi: 10.1186/s13244-025-02184-2 (PMC12834855; doi:10.1186/s13244-025-02184-2)
Supplement: Supplementary file 1 — ELECTRONIC SUPPLEMENTARY MATERIAL [file 13244_2025_2184_MOESM1_ESM.pdf]

Contrast-enhanced CT-based radiomics for predicting visceral pleural invasion in early-stage non-small cell lung cancer

ELECTRONIC SUPPLEMENTARY MATERIAL

Insights Imaging (2025) Luo QY, Li HT, Zheng YT, et al.

Table S1 Baseline characteristics in the training, validation and testing sets

	Training set (n=264)	Validation set (n=114)	Testing set (n=145)	p value
Gender (male)	114(43.2%)	50(43.9%)	67(46.2%)	0.838
Age (years), median (IQR)	59[52,65]	59[53,67]	60[54.67]	0.553
Smoking	62(23.5%)	20(17.5%)	32(22.1%)	0.437
Relationship to the pleura				0.518
Type I	41(15.5%)	12(10.5%)	17(11.7%)	
Type II	38(14.4%)	19(16.7%)	19(13.1%)	
Type III	27(10.2%)	11(9.6%)	15(10.3%)	
Type IV	22(8.3%)	18(15.8%)	16(11.0%)	
Type V	136(51.5%)	54(47.4%)	78(53.8%)	
Density				0.013*
pGGO	29(11.0%)	15(13.2%)	18(12.4%)	
mGGO	151(57.2%)	61(53.5%)	58(40.0%)	
solid	84(31.8%)	38(33.3%)	69(47.6%)	
Maximum diameter (mm), median (IQR)	19[15,22]	18[16,22]	19[14,24]	0.721
Spiculation	130(49.2%)	59(51.8%)	77(53.1%)	0.739
Lobulation	73(27.7%)	33(28.9%)	48(33.1%)	0.508
Lymphadenopathy	28(10.6%)	8(7.0%)	21(14.5%)	0.157
Presence of a solid portion in contact with the pleura	133(50.4%)	54(47.4%)	77(53.1%)	0.656
Tumor location				0.090
Left upper lobe	63(23.9%)	25(21.9%)	25(17.2%)	
Left lower lobe	32(12.1%)	11(9.6%)	25(17.2%)	
Right upper lobe	87(33.0%)	45(39.5%)	43(29.7%)	
Right middle lobe	32(12.1%)	5(4.4%)	17(11.7%)	
Right lower lobe	50(18.9%)	28(24.6%)	35(24.1%)	
Involved pleura				0.250
Non-interlobar fissure pleura	162(61.4%)	71(62.3%)	85(58.6%)	
Interlobar fissure pleura	59(22.3%)	22(19.3%)	24(16.6%)	
both	43(16.3%)	21(18.4%)	36(24.8%)	
Cavity	24(9.1%)	7(6.1%)	15(10.3%)	0.481
Pleural thickening	118(44.7%)	51(44.7%)	73(50.3%)	0.512
Pleural effusion	0(0.0%)	0(0.0%)	1(0.7%)	0.492
Calcification	6(2.3%)	1(0.9%)	6(4.1%)	0.271

* p value<0.05.

IQR, interquartile range; pGGO, pure ground-glass opacity; mGGO, mixed ground-glass opacity.

Table S2 Analysis of inter-reader consistency for CT features

CT features	Agreement	Kappa(95%CI)	Interpretation
Relationship to the pleura	0.90	0.862 [0.714, 1.000]	Almost perfect
Density	0.92	0.861 [0.812, 0.997]	Almost perfect
Involved pleura	0.94	0.886 [0.665, 1.000]	Almost perfect
Spiculation	0.90	0.775 [0.590, 0.960]	Substantial
Lobulation	0.92	0.783 [0.583, 0.982]	Substantial
Lymphadenopathy	0.98	0.790 [0.391, 1.000]	Substantial
Presence of a solid portion in contact with the pleura	0.94	0.876 [0.740, 1.000]	Almost perfect
Pleural thickening	0.90	0.786 [0.610, 0.962]	Substantial

Table S3 Univariate and multivariate analysis for patients in the training set

Variables	Univariate		Multivariate	
	OR (95%CI)	p value	OR (95%CI)	p value
Relationship to the Pleura				
Type I	Ref.			
Type II	2.83 (1.02-8.48)	0.051	1.97 (0.62-6.64)	0.256
Type III	8.26 (2.79-27.09)	<0.001*	4.88 (1.36-19.05)	0.018*
Type IV	2.78 (0.85-9.4)	0.093	6.24 (1.16-37.09)	0.037*
Type V	2.48 (1.07-6.49)	0.045*	1.84 (0.52-6.93)	0.352
Density				
pGGO	Ref.			
mGGO	3.92 (1.1-25.09)	0.071	2.31 (0.39-20.54)	0.395
solid	27 (7.38-175.04)	<0.001*	8.80 (1.28-88.12)	0.039*
Maximum diameter	1.14 (1.09-1.21)	<0.001*	1.08 (1.01-1.16)	0.019*
Spiculation	3.67 (2.16-6.37)	<0.001*	1.70 (0.84-3.45)	0.140
Lobulation	4.92 (2.79-8.84)	<0.001*	1.67 (0.77-3.59)	0.190
Lymphadenopathy	2.81 (1.27-6.35)	0.011*	1.67 (0.65-4.42)	0.290
Presence of a solid portion in contact with the pleura	1.79 (1.08-3.01)	0.026*	1.00 (0.38-2.69)	0.998
Tumor location				
Left upper lobe	Ref.			
Left lower lobe	1.14 (0.44-2.84)	0.787		
Right upper lobe	1.85 (0.93-3.75)	0.082		
Right middle lobe	1.5 (0.6-3.69)	0.378		
Right lower lobe	1.07 (0.47-2.42)	0.868		
Involved Pleura				
Non-interlobar fissure pleura	Ref.			
Interlobar fissure pleura	0.53 (0.26-1.02)	0.066	0.94 (0.39-2.21)	0.891
both	1.22 (0.61-2.42)	0.563	1.01 (0.43-2.33)	0.986
Cavity	1.13 (0.46-2.66)	0.775		
Pleural thickening	1.49 (0.89-2.48)	0.127		
Pleural effusion	NA	NA		
Calcification	0.37 (0.02-2.32)	0.364		

*, statistically significant. OR, odds ratio. CI, confidence interval. Ref.,reference. pGGO, pure ground-glass opacity. mGGO, mixed ground-glass opacity.

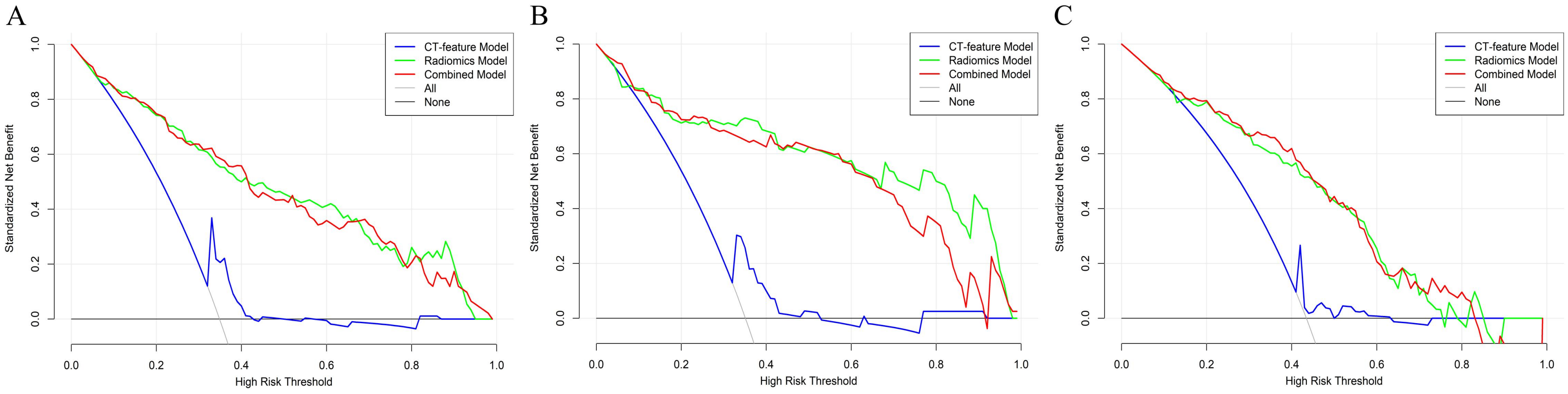

Fig S1: Clinical utility of the three models using DCA. DCA, Decision Curve Analysis
